# Air Pollutant Patterns and Human Health Risk following the East Palestine, Ohio, Train Derailment

**DOI:** 10.1021/acs.estlett.3c00324

**Published:** 2023-07-12

**Authors:** Oladayo Oladeji, Mariana Saitas, Toriq Mustapha, Natalie M. Johnson, Weihsueh A. Chiu, Ivan Rusyn, Allen L. Robinson, Albert A. Presto

**Affiliations:** † Department of Mechanical Engineering, Carnegie Mellon University, Pittsburgh, Pennsylvania 15213, United States; ‡ Department of Environmental and Occupational Health, Interdisciplinary Faculty of Toxicology, Texas A&M University, College Station, Texas 77843, United States; § Department of Veterinary Physiology and Pharmacology, Interdisciplinary Faculty of Toxicology, Texas A&M University, College Station, Texas 77843, United States

**Keywords:** air toxics, hazardous air pollutants, VOCs, disaster response research, mobile monitoring

## Abstract

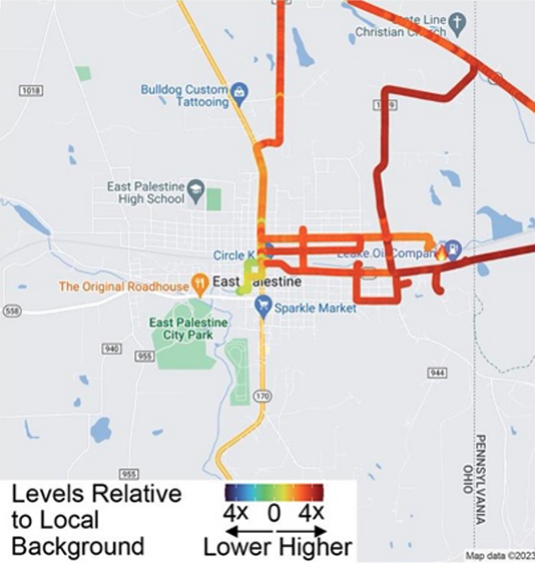

On February 3, 2023, a train carrying numerous hazardous chemicals derailed in East Palestine, OH, spurring temporary evacuation of residents and a controlled burn of some of the hazardous cargo. Residents reported health symptoms, including headaches and respiratory, skin, and eye irritation. Initial data from U.S. Environmental Protection Agency (EPA) stationary air monitors indicated levels of potential concern for air toxics based on hazard quotient calculations. To provide complementary data, we conducted mobile air quality sampling on February 20 and 21 using proton transfer reaction-mass spectrometry. Measurements were taken at 1 s intervals along routes designed to sample both close to and farther from the derailment. Mobile air monitoring indicated that average concentrations of benzene, toluene, xylenes, and vinyl chloride were below minimal risk levels for intermediate and chronic exposures, similar to EPA stationary monitoring data. Levels of acrolein were high relative to those of other volatile organic compounds, with spatial analyses showing levels in East Palestine up to 6 times higher than the local rural background. Nontargeted analyses identified levels of additional unique compounds above background levels, some displaying spatiotemporal patterns similar to that of acrolein and others exhibiting distinct hot spots. These initial findings warrant follow-up mobile air quality monitoring to characterize longitudinal exposure and risk levels.

## Introduction

Substantial releases of hazardous volatile organic compounds (VOCs) into the atmosphere following both natural and anthropogenic disasters are increasingly recognized, as exemplified by the changes in hazardous air pollutant (HAP) emissions along the Texas Gulf Coast after Hurricane Harvey^1^ in 2017 and in eastern North Carolina following Hurricane Florence^2^ in 2018. In the aftermath of the Intercontinental Terminals Company fire in 2019 in Deer Park, TX, high levels of benzene, a known human carcinogen, necessitated a shelter in place as a public health measure.^3^

Another example of a massive release of VOCs due to an anthropogenic disaster is the event that occurred on February 3, 2023, when a freight train carrying many hazardous chemicals, including vinyl chloride, derailed in the village of East Palestine, OH, ∼50 miles northwest of Pittsburgh, PA (for the train manifest, see Figure S3). Initially, nearly 2000 of the town’s ∼4700 residents were told to evacuate from within one mile of the crash site due to air quality concerns. Fearing a chemical explosion of the tank cars with vinyl chloride, authorities initiated a controlled release and burn on February 6, and the evacuation zone was expanded to a 1 × 2 mile area because additional air toxics were released. By February 8, the evacuation order was lifted, but reports of air quality-related health effects persisted. According to a health survey^4^ conducted within weeks of the derailment, local residents reported a range of sensory irritation and psychosomatic symptoms consistent with exposures to VOCs, including headache (74%), anxiety (64%), cough (61%), fatigue/tiredness (58%), and irritation, pain, or burning of skin (52%). The U.S. Environmental Protection Agency (EPA) set up stationary air monitor(s) to inform local ambient air quality, and the preliminary data have been posted online.^5^

Exposure assessment for air pollution is a critical input into risk assessment and management.^6^ Air quality monitoring in the aftermath of a disaster is an especially challenging task. There are three prominent challenges in research for disaster response for air toxics: the lack of appropriate local background (i.e., predisaster) levels, the low sensitivity of the instrumentation that is often deployed for rapid response, and the paucity of long-term measurements of air quality beyond the immediate weeks after an event.

One typical source of background information is the routine EPA observation of hazardous air pollutants (HAPs), also termed air toxics, through a network of stationary air monitors as part of the National Air Toxics Trends Station (NATTS) Network.^7^ The current network includes 26 sites (21 urban and five rural) across the United States. There are typically >100 HAPs monitored at each NATTS, although only 19 of those are formally required. Target HAPs include VOCs, carbonyls, PM_10_ metals, and polycyclic aromatic hydrocarbons (PAHs). Another example of the information that is used for evaluation of air toxics across the United States is the EPA’s Air Toxics Screening Assessment or AirToxScreen. This tool provides census tract-level estimates of concentrations of air toxics based on the National Emissions Inventory (data on emissions from point, nonpoint, and mobile sources, biogenic sources, and fires) and two air quality models: the American Meteorological Society/Environmental Protection Agency Regulatory Model (AERMOD) atmospheric dispersion model and the Community Multiscale Air Quality (CMAQ) photochemical model.^8^ However, confidence in using NATTS, AirToxSceen, or site-specific data collected using typical field-deployable analytical approaches for risk management is often hindered by both model uncertainty and method detection limits. This is especially relevant in disaster response because most instruments used by government agencies and other parties responding to disasters suffer from a lack of sensitivity, resulting in method detection limits that are near or greater than corresponding health-protective exposure thresholds.^7^

Repeated measures with improved spatial and temporal resolution are needed during disaster response to assess both short- and long-term consequences of HAP releases. Moreover, nontargeted analyses for identification of chemicals that may be present beyond a predetermined range of analytes (i.e., targeted analyses) constitute a current gap in rapid response and emergency scenarios.^9,10^ Therefore, to provide both context and complementary data to EPA stationary monitoring during initial phases of disaster response and recovery of the East Palestine disaster, we conducted mobile air monitoring using a highly sensitive nontargeted approach of proton transfer reaction time-of-flight (PTR-ToF) mass spectrometry^11^ to characterize spatial and temporal patterns of VOCs near the site of a derailment and a subsequent controlled burn.

## Materials and Methods

Stationary air monitoring data were downloaded from the EPA.^5^ The hazard quotient (HQ) for East Palestine data was determined by dividing the median and highest reported concentrations by the EPA reference concentration (RfC) values (Table S3), which are levels considered to be without appreciable risk of deleterious effects over lifetime exposure.^12^ For comparison, HQ values were also determined for median and highest county-level ambient air concentration levels in the United States, state of Ohio, and Columbiana County, where East Palestine is located, using the most recent EPA AirToxScreen data release.^8^ HQ values of <1 indicate little concern for a single chemical, and HQ values of <0.1 indicate little concern for multiple chemicals present at the same time. In addition, both EPA data and our data were compared to the Agency for Toxic Substances and Disease Registry (ATSDR) intermediate (15 days to 1 year) and chronic (>1 year) minimal risk levels (MRLs).

Mobile air monitoring measurements were conducted by using the Carnegie Mellon University (CMU) mobile air quality laboratory. The mobile laboratory is an instrumented Nissan 2500 cargo van, previously described by Li et al.^13,14^ All instruments in the mobile laboratory were powered by a 110 V, 60 Hz alternator coupled to the van’s engine. A pair of 0.5 in. outside diameter stainless steel tubes carried the samples from the roof of the van (∼3 m above ground level, at the front of the van) to instruments as well as a mechanical backing pump. Previous deployments of this mobile platform over the previous decade indicate that self-sampling is not a concern.^15−17^ We saw no indication of self-sampling during the testing and sampling periods.

The data we report here rely on measurements made with an Ionicon Analytik (Innsbruck, Austria) 4000 PTR-ToF mass spectrometer. The PTR-ToF operations and analysis details are described in the Supporting Information. Briefly, the mobile sampling strategy followed our previous field sampling campaigns.^15^ The van was driven along public roads in East Palestine at approximately 20–25 mph. The PTR-ToF collected data at 1 Hz; this yielded a spatial resolution of ∼11 m when PTR-ToF data were merged with GPS coordinates. Measurements were collected on February 21 and 22, 2023. On both days, sampling was conducted in locations upwind and downwind of the train derailment site. On February 20, 2023, the PTR-ToF was operated in hydronium mode (H_3_O) from approximately 11:30 am until 8:00 pm. On February 21, 2023, the PTR-ToF was operated in oxygen (O_2_) mode, which provides improved sensitivity for chlorinated compounds from approximately 12:00 pm until 7:30 pm.

## Results

**Figure 1:**
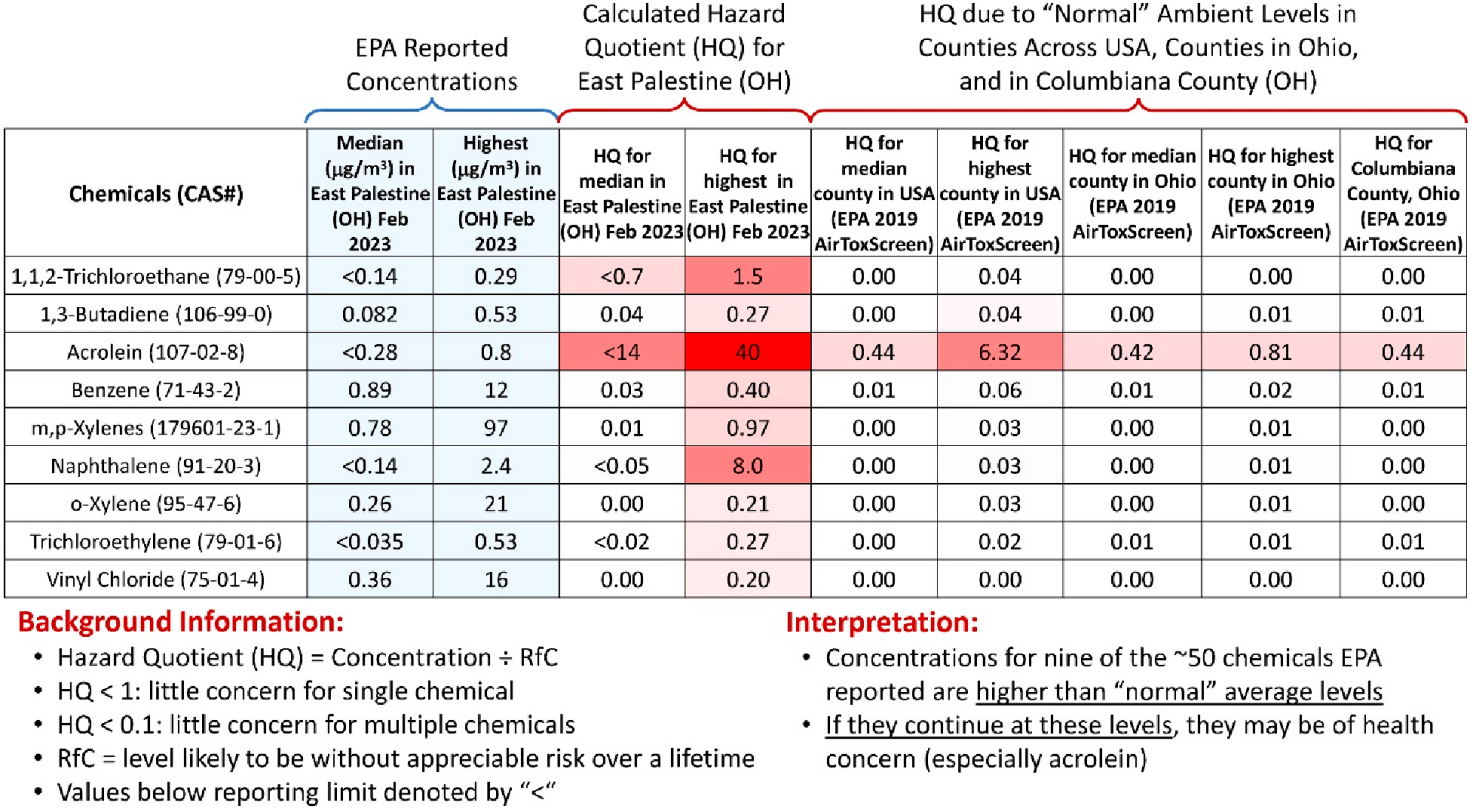
Comparison of hazard quotients (HQs) based on EPA stationary monitoring data in East Palestine, OH, with those determined from the most recent (2019) EPA National Air Toxics estimates. All HQ values were compared to the RfC values from the EPA Superfund RSL Table (November 2022 version). For some concentrations in East Palestine, levels of nine of ∼50 chemicals EPA reported were higher than “normal”. If these levels persist, then they may be of health concern (especially acrolein).

The preliminary data reported^5^ by EPA stationary air monitoring in East Palestine indicated that concentrations for nine of the approximately 50 chemicals measured were relatively high in comparison to the levels considered safe for lifetime exposure (Figure 1). Acrolein had the greatest HQ values for both the median (14.0) and highest (40) measurements. Notably, however, the detection limit for acrolein in the EPA data release^5^ for East Palestine is much higher than the RfC, so although the median measured value in East Palestine was below the reporting limit, these data cannot ensure that acrolein levels are below those of long-term health concern. Additionally, 1,1,2-trichloroethane and naphthalene had calculated HQ values of >1.0 for the highest values measured in East Palestine, though median levels were lower. The remaining VOCs, including 1,3-butadiene, benzene, xylenes, trichloroethylene, and vinyl chloride, had calculated HQ values of >0.1 for the highest values measured in East Palestine. Overall, if ambient levels persisted for these chemicals, they could pose health concerns, either individually (e.g., acrolein, a known respiratory irritant) or cumulatively. Thus, subsequent, spatiotemporal analysis was pertinent.

**Figure 2:**
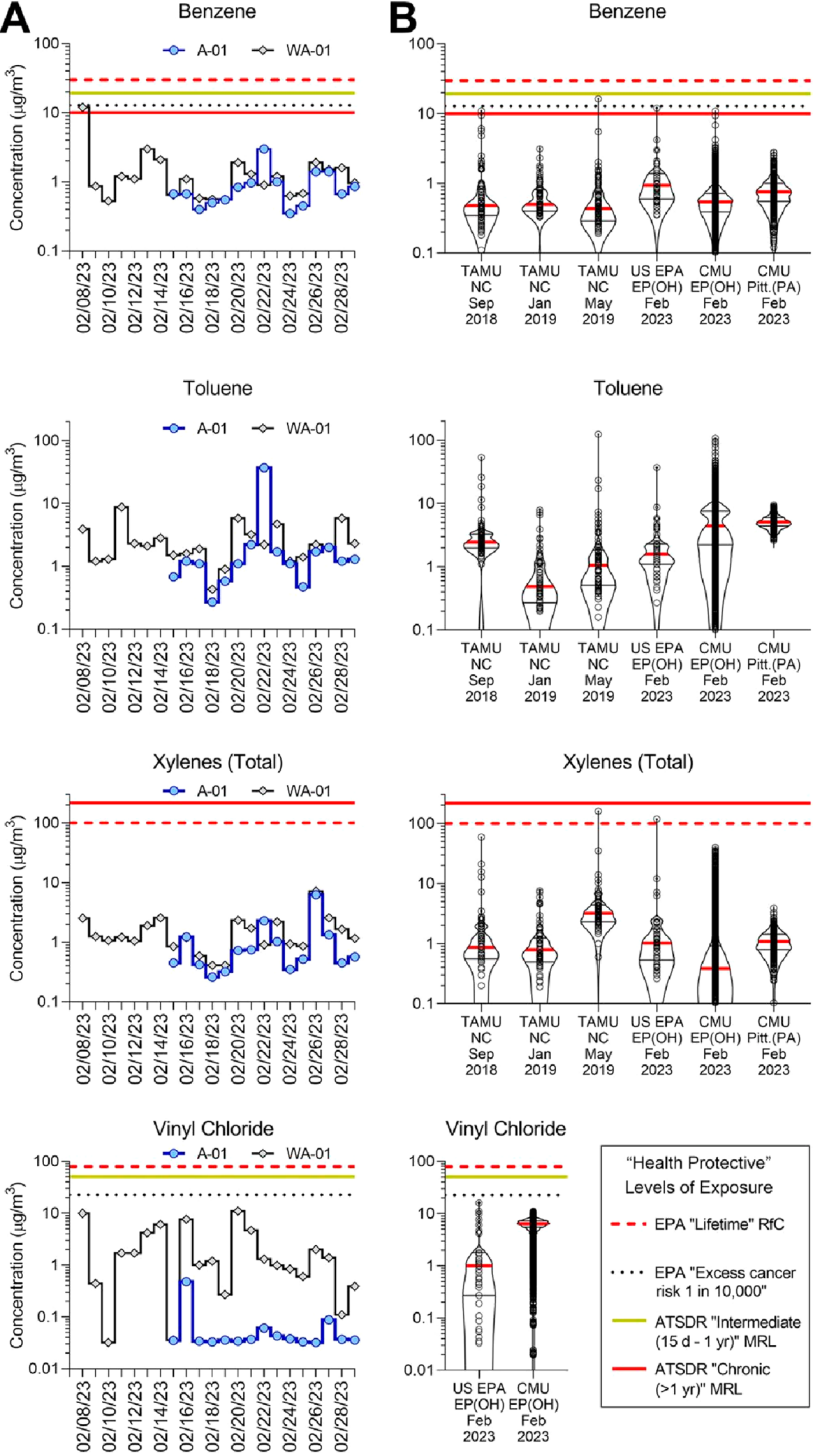
(A) Concentrations of benzene, toluene, xylenes, and vinyl chloride in East Palestine reported by EPA stationary monitor(s) from February 8 to 22. Site A-01 was in the center of East Palestine, and site WA-01 was near the derailment site. (B) Violin plots provide context from past mobile monitoring by Texas A&M University (TAMU) after Hurricane Florence in eastern North Carolina (September 2018 through May 2019). EPA stationary monitoring data from February compared with mobile monitoring data from Carnegie Mellon University (CMU) mobile monitoring in East Palestine and Pittsburgh: thick red line, median; thin black lines, quartiles; dots, individual sample data, in context with health-protective levels (see Table S3); yellow line, ATSDR intermediate (15 days to 1 year MRL); solid red line, ATSDR chronic (>1 year) MRL; dashed red line, EPA lifetime RfC; dashed black line, EPA excess cancer risk 1 in 10 000.

Temporal data from EPA monitoring sites A-01 (in the center of East Palestine) and WA-01 (near the derailment site) from February 8 to March 1, 2023 (Figure 2A), indicated sustained decreases in ambient concentrations of benzene, toluene, and xylenes. All were under different types of health-protective levels of exposure. While vinyl chloride levels varied during this time, all concentrations were also under established levels of health concern. To contextualize these measurements to another environmental disaster scenario, we compared concentrations with those from our previous mobile monitoring^2^ in response to Hurricane Florence in eastern North Carolina (Figure 2B). Median concentrations of benzene, toluene, and xylenes were similar to median concentrations detected by mobile monitoring in East Palestine and in Pittsburgh. Moreover, median concentrations were below health-protective levels of exposure and were similar to EPA stationary monitoring data. Vinyl chloride, typically not present in ambient air and one of the chemicals onboard the train that derailed, showed higher median levels for mobile monitoring data. This may be explained by the enhanced spatial variability and shorter sampling window (i.e., only 1 day). Even so, levels were also under established levels of health concern.

**Figure 3:**
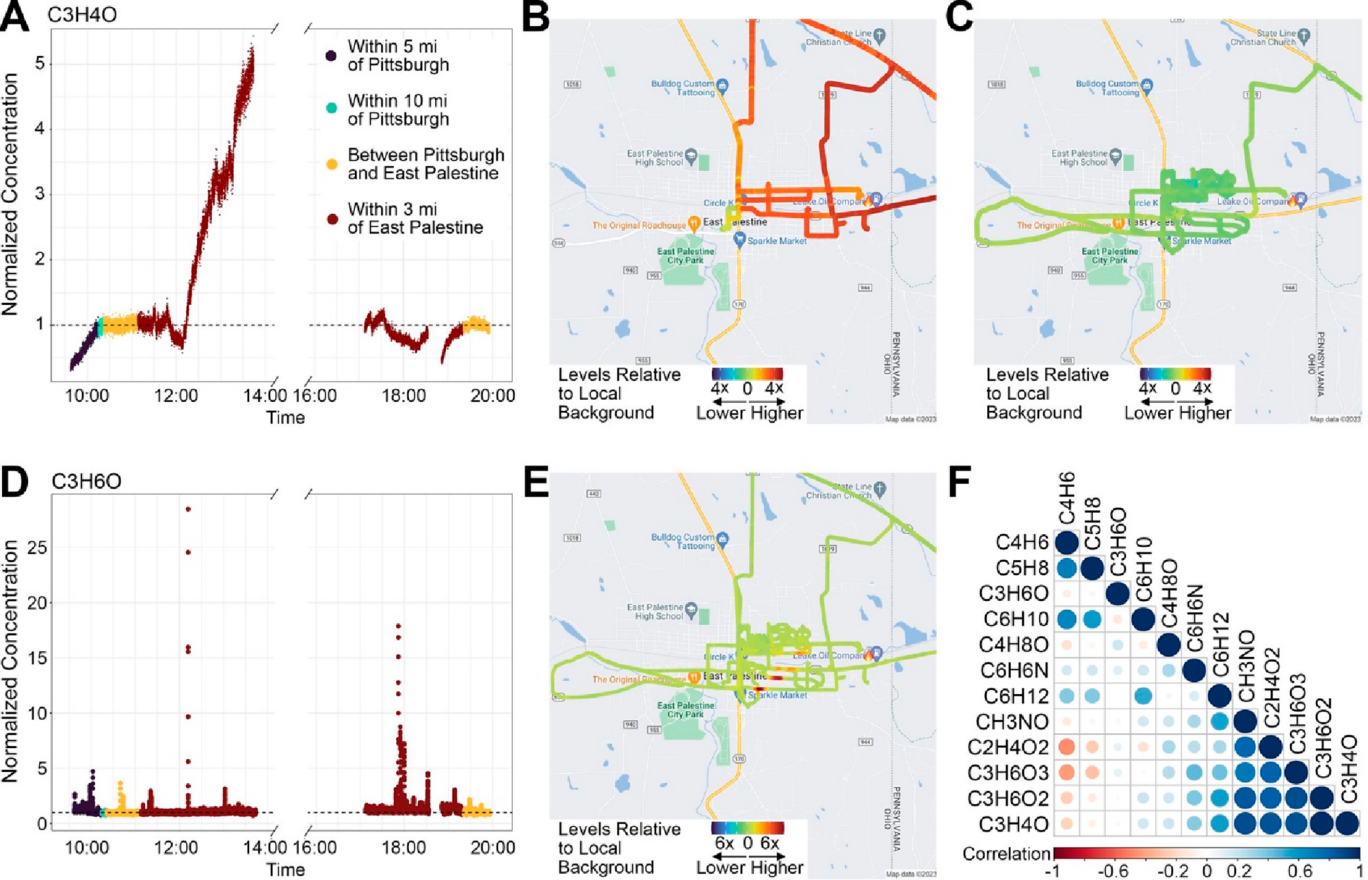
Acrolein (C_3_H_4_O) concentrations during mid-day (10:00 to 14:00) and evening (17:00 to 20:00) on February 20, 2023, each normalized to the local rural background collected between Pittsburgh and East Palestine, OH. (A) Temporal distribution and (B and C) spatial distribution of acrolein concentrations. (D and E) Concentrations of a chemical with a mass of 58.080, identified as C_3_H_6_O, showing temporal and spatial “hot spots” in East Palestine. (F) Correlations among 12 unique molecular masses revealed by analysis of nontargeted data in the hydronium mode. Levels of several (CH_3_NO, C_2_H_4_O_2_, C_3_H_6_O_3_, and C_3_H_6_O_2_) were above local background levels and displayed temporal and spatial patterns similar to those of acrolein (C_3_H_4_O). Meteorological information for this sampling day is summarized in Figure S4.

Consistent with the EPA stationary sampling data, levels of acrolein measured through mobile air monitoring were high relative to those of other volatile compounds detected. Temporal analysis showed higher levels in the mid-day sampling versus the evening sampling (Figure 3A). Spatial analysis showed that the level of acrolein was ≤6 times higher than the local rural background near the train derailment site (Figure 3B). Levels were largely at or below the background in the evening sampling across the sampled area (Figure 3C).

Nontargeted data analysis revealed additional unique species. The levels of several were above local background levels and displayed temporal and spatial patterns similar to those of acrolein, which was confirmed by correlation analysis (Figure 3F). These included compounds CH_3_NO (formamide), C_2_H_4_O_2_, C_3_H_6_O_2_, C_3_H_6_O_3_, C_11_H_20_O_2_ (ethyl hexyl acrylate), and C_17_H_12_O_2_ (butyl acrylate); all were detected as the protonated parent ion as described in the Supporting Information. These compounds are a mix of species carried on the train (e.g., butyl acrylate) and potential oxidation products formed either during combustion or via atmospheric chemistry. C_2_H_4_O_2_ was identified as possibly acetic acid or methyl formate; however, the PTR-ToF could not distinguish between these. C_3_H_6_O_2_ could be several compounds that have the same exact mass, including (1) acetic acid, methyl ester; (2) formic acid, ethyl ester; or (3) propanoic acid. C_3_H_6_O_3_ was identified as carbonic acid and dimethyl ester. Other species, such as C_3_H_6_O, exhibited distinct hot spots above background levels in different parts of the sampling area (Figure 3D,E). This species, identified as the protonated ion at an exact mass of *m*/*z* 58.080, could represent many potential chemicals, including (1) acetone, (2) methoxy ethene, (3) oxetane, (4) propanal, or (5) propylene oxide. Several other compounds were detected, however, with no clear spatial patterns, for instance, C_4_H_8_O, C_5_H_8_, or C_6_H_10_. In all cases, the concentrations of these compounds were well below health relevant exposure limits.

## Discussion

Following the train derailment, chemical spill, and controlled burn, the EPA began targeted stationary air monitoring. On the basis of our analysis of HQs determined for East Palestine, several chemicals posed higher risk than normal background levels in the United States and Ohio. Importantly, these included several known respiratory irritants and human carcinogens. Our initial calculations provided the rationale for conducting mobile monitoring in East Palestine. Data from our mobile air monitoring in East Palestine provide an example of the utility of highly sensitive, nontargeted VOC measurements with enhanced spatial resolution for characterizing air quality postdisaster, particularly to investigate two key questions. (1) How representative are stationary monitoring data in space and time? (2) Have targeted analyses missed any VOCs with increased levels?

With respect to the first question, we found that levels of targets benzene, toluene, and xylenes were largely similar to stationary monitoring data. Additionally, these levels were similar to baseline levels in the United States and to levels from postdisaster mobile monitoring we previously conducted in 2018–2019 of emissions after Hurricane Florence and were all below long-term health thresholds. These chemicals are ubiquitous VOCs with large mobile source contributions. Similarly, while the level of vinyl chloride, one of the chemicals carried on the train and a known human carcinogen, was initially increased, measurements over time from both stationary and mobile monitoring indicated levels below long-term health thresholds. Importantly, for many of these compounds, including oxidation products of vinyl chloride, there is still a lack of hazard data for acute end points, especially in sensitive subpopulations, which warrants additional research. Reports of symptoms in East Palestine indicated a portion of the population experienced headaches, nausea, cough, bloody noses, and respiratory, skin, and eye irritation. Additional information from health surveys will provide further context for adverse health effects.

By contrast, elevated levels of acrolein were identified by both stationary and our mobile monitoring data, with targeted analyses from the EPA showing some levels substantially above long-term health thresholds. Acrolein is a common combustion product and a known respiratory irritant. Breathing low levels is linked with eye watering, burning of the nose and throat, and decreased breathing rates. Experimental studies show inhalation causes irritation of the nasal cavity, decreased breathing rates, and damage to the lining of the lung, as well as pathological lesions and nasal tumors with long-term chronic exposure. Additionally, our mobile sampling data indicated substantial spatial and temporal variation in acrolein that could not be characterized through stationary sampling.

With respect to the question of whether targeted analyses “missed” anything, our nontargeted mobile sampling found numerous other chemicals with increased levels in East Palestine compared to the local rural background. A number of these had spatiotemporal patterns that correlated highly with that of acrolein, suggesting a common source. Although only putatively identified, a number of these compounds have also been linked to respiratory effects. For instance, exposure to formamide has been linked with irritation of the eyes and skin, drowsiness, and nausea,^18^ and methyl formate exposure has been linked with numerous symptoms, including irritation of the eyes and nose, chest tightness, dyspnea, and central nervous system depression.^18^ Several other compounds exhibited distinct hot spot patterns with peaks ≤10 times above the background, but characterizing their persistence would require additional sampling.

These initial findings support the need for continued mobile monitoring to characterize the air quality impacts of the East Palestine train derailment, especially as cleanup activities may result in resuspension and revolatilization of contaminants from the soil. More broadly, this study illustrates that the ability of highly sensitive, nontargeted mobile monitoring to detect known and unknown VOCs can serve as a complement to the targeted and stationary monitoring typically deployed, facilitating characterization of the impacts of disasters on air quality and ultimately better protecting public health.

## Supporting Information Available

Additional details of PTR-ToF operation, calibration, and data analysis; health relevant reference levels for comparison to ambient concentrations; train manifest; and meteorological data (PDF)
