# Supplementary material for: Air Pollutant Patterns and Human Health Risk following the East Palestine, Ohio, Train Derailment
Source: Environ Sci Technol Lett. 2023 Jul 12;10(8):680–5. doi: 10.1021/acs.estlett.3c00324 (PMC10413936; doi:10.1021/acs.estlett.3c00324)
Supplement: Supplementary file 1 — ez3c00324_si_001.pdf [file ez3c00324_si_001.pdf]

Supplemental Information

Air pollutant patterns and human health risk following the East Palestine, Ohio train derailment

Oladayo Oladeji, Mariana Saitas, Toriq Mustapha, Antonios Tasoglou, Natalie M. Johnson, Weihsueh A. Chiu, Ivan Rusyn, Allen L. Robinson, Albert A. Presto*

This document contains 10 pages, 4 figures, and 3 tables.

1. Proton transfer reaction time-of-flight reaction (PTR-ToF) mass spectrometer operation

The PTR-ToF detects volatile organic compounds (VOCs) through "soft" ionization. Thus, most analytes are detected as the parent ion with minimal fragmentation. The Ionicon, Inc. 4000 time-of-flight mass spectrometer has a mass resolution of 10,000. This means that the PTR-ToF-MS can separate ions that have the same nominal mass but different amounts of C, H, O, and heteroatoms.

The PTR-ToF was operated in two modes. In the traditional hydronium mode, ionization is achieved via proton transfer (hence the instrument name):

$$H_3^+O + X \rightarrow XH^+ + H_2O$$

Here, X is the analyte of interest. This mode is sensitive to species that have a higher proton affinity than water, which includes a wide array of atmospheric VOCs but crucially does not include small aliphatic molecules like methane and ethane that would otherwise dominate the signal.

The PTR-ToF can also be operated in oxygen mode. Here the charge carrier is O_2^+, and ionization is via direct charge transfer.

$$O_2^+ + X \rightarrow X^+ + O_2$$

Oxygen mode is significantly more sensitive to chlorinated organic compounds that have low proton affinities than hydronium mode.

At the start and end of each day of sampling, we sampled zero air with the instrument. This was used to determine the instrument baseline, which was subsequently subtracted during analysis. Additionally, a multi-point calibration was conducted each night with a 16-component calibration mixture (Table 1). The calibration standard was dynamically diluted with zero air during each nightly calibration.

Table S1. Composition of the PTR-ToF calibration gas.

Formula	Name	Exact mass (MH+)	Concentration in standard (ppb)	k-rate x10^9 cm^3 $molecule^{-1}$ s^{-1}
Proton mode species				
CH4O	Methanol	32.026215	2126	2.185
C2H3N	Acetonitrile	41.026549	2316	4.74
C3H6O	Acetone	58.041865	2260	3.155
C5H8	Isoprene	68.0626	2229	1.966
C4H6O	Methyl Vinyl Ketone	70.041865	2217	3.188
C4H8O	2- Butanone	72.057515	2256	3.041
C6H6	Benzene	78.04695	2239	1.925
C7H8	Toluene	92.0626	2242	2.136
C8H8	Styrene	104.0626	2271	2.33
C8H10	p-Xylene	106.07825	2210	2.191
C9H12	1,3,5-Trimethylbenzene	120.0939	2221	2.4
C10H14	1,2,3,5 Tetramethylbenzene	134.10955	2319	2.5
Oxygen mode species				
C2H3Cl	Vinyl Chloride	61.992328	2171	
C2H2Cl2	1,2-Dichloroethylene	96.95671	2254	
C2HCl3	Trichloroethylene	129.914383	2233	
C2Cl4	Tetrachloroethylene	165.872461	2193	

2. Proton transfer reaction time-of-flight reaction (PTR-ToF) mass spectrometer data analysis

Ion abundance was determined via peak integration in the PTR-MS Viewer 3.22 software (Figure 1). Target analysis uses a predefined peak table to select ions for integration. In cases of isobaric ions, a multi-peak fit was applied to separate the (clearly distinct) peaks. Ion abundance was also adjusted for ion transmission efficiency in the PTR-ToF. The transmission efficiency curve was generated daily using our calibration mixture.

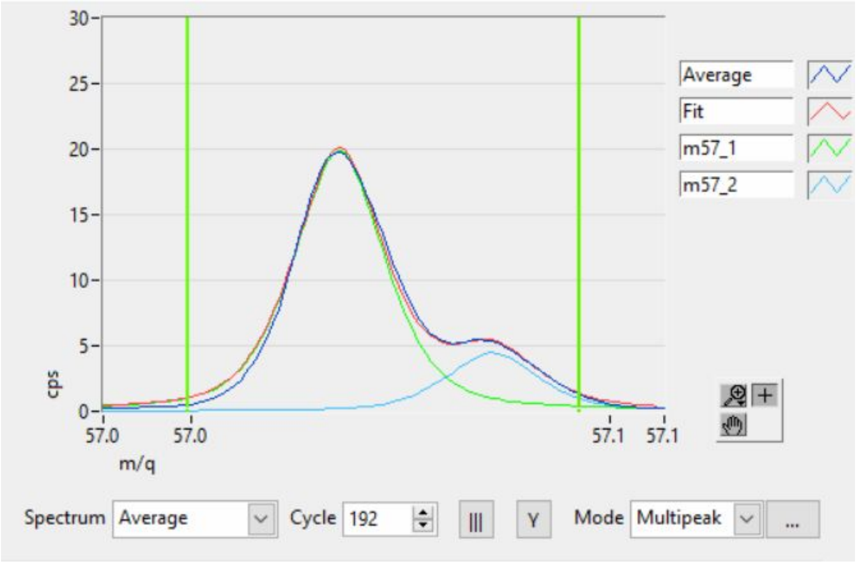

Figure S1. Peak integration of raw data. There are two distinct peaks with a nominal mass of *m/z* 57: $C_3H_4OH^+$ and $C_4H_9^+$. The oxygenated peak has a slight negative mass defect (actual mass < nominal mass) because of the presence of oxygen atoms, whereas the unoxygenated ion has a positive mass defect. The cyan and green traces show integration of these separate ions.

Two methods were used to convert ion abundances to species concentration. The first directly considered the kinetics of the proton transfer reaction:[1]

$$\frac{d[XH^+]}{dt} = k[H_3O][X]$$

For a fixed reaction time (which exists in the PTR-ToF), the above reduces to an algebraic expression. If k is known, then the concentration of analyte [*X*] can be computed. We used this approach for compounds measured in hydronium mode. Many measured k values are published in the literature and therefore readily available.[2] The reaction rate k can also be calculated from molecular properties following the method of Sekimoto et al.[3] When k is not published, a default value of 2×10^{-9} cm^{-3} molecule s^{-1} is commonly used.

The other method applied was to compute [*X*] using a calibration factor. These calibration factors are expressed in units of ppb per normalized ion counts (ppb/ncps).[4] In this approach, the raw ion signal for each ion is normalized by the primary ion signal (either H_3O^+ or O_2^+ depending on the instrument mode). We use this approach to determine concentrations of chlorinated species, such as vinyl chloride, measured in oxygen mode.

3. Target species and data quality metrics

Table 2 shows species identified during target analysis. For each target species, we defined the minimum detection limit (MDL) as three times the standard deviation of the signal measured while sampling zero air.

Table S2. Species reported in targeted analysis. [a]Units of *k* are cm^{-3} $molecule^{-1}$ s^{-1}. [b]Acrolein sensitivity is discussed in the following section. [c]Reported as the sum of all xylene isomers and ethyl benzene at *m/z* 107.

**Species**	**CASRN**	**Mode**	**HR ion used**	**Minimum detection limit**	***k*[a] x10[9]**	**Sensitivity (ncps/ppb)**
Acetaldehyde	75-07-0	H+	44.99329	1.17	3.12	
Acrolein	107-02-8	H+	57.03604	1 cps[b]	N/A[b]	
Propanal + Acetone		H+	59.05214	0.56	3.155	
Methanol	67-56-1	H+	33.02956	2.07	2.185	
m-Xylene[c]	108-38-3	H+	107.08553	0.24	2.191	
Toluene	108-88-3	H+	93.06488	1.78	2.136	
Naphthalene	91-20-3	H+	129.06987	0.15	2.59	
Acetonitrile	75-05-8	H+	42.02802	0.27	4.74	
Dibutyl 1,2-benzenedicarboxylate	84-74-2	H+	279.19909	0.047	2	
Vinyl chloride	75-01-4	O2+	61.994	1.04		3.04
Benzene	71-43-2	H+	79.05423	0.14	1.925	

While the PTR-ToF can separate isobaric ions with different composition, it cannot separate species with identical composition. Thus, *m/z* 59.05 represents the sum of acetone and propanal (C_3H_6O) and *m/z* 107.08 represents all isomers of xylene and ethyl benzene with the formula C_8H_{10}.

3.1 Quantification of acrolein

Acrolein was identified as a priority component because of high concentrations in the canister samples collected by the EPA. Several previous studies have used PTR-ToF to quantify acrolein, primarily in emissions from biomass burning. There is wide variation in the published calibration factors for acrolein.

Brilli et al.[5] used the traditional default *k* of $2x10^{-9}$ cm^{-3} $molecule^{-1}$ s^{-1}. They did not directly calibrate for acrolein. Sekimoto et al.[3] presented a method for calculating *k*. They also performed experimental measurements to evaluate their predictions. The calculated *k* for acrolein to be $3.1x10^{-9}$ cm^{-3} $molecule^{-1}$ s^{-1}. However, they also observed that the sensitivity for acrolein and other small, oxygenated molecules was only ~40% of the expected sensitivity based on *k* alone. They attributed this to delocalization of electrons in the conjugated doubled bonds in acrolein. Schieweck et al.[6] measured acrolein in an indoor environment and determined a *k* of $3.55x10^{-9}$ cm^{-3} $molecule^{-1}$ s^{-1}.

Two other papers reported acrolein sensitivity instead of *k*. Lastly, Koss et al.[7] reported a sensitivity of 38.73 ncps/ppb for acrolein. However, those experiments used a slightly different version of the PTR-ToF. Stockwell et al.[8] reported a sensitivity nearly an order of magnitude lower.

We did not have a calibration standard for acrolein. Given the wide range in published acrolein calibrations, we do not report acrolein concentration. Instead, we report in Table 2 the minimum detection limit in terms of counts per second (cps). Analysis of acrolein data compares signals (e.g., between Pittsburgh and East Palestine) but does not report concentrations directly.

3.2 Calibration checks

We evaluated instrument performance each day by comparison to a calibration mixture. We evaluated the slope of the linear correlation between the supplied and measured concentrations, as well as the linearity of the calibration. Our calibrations demonstrate excellent linearity ($R^2 > 0.99$).

Figure 2 shows calibration results for vinyl chloride and benzene. The left panel shows the determinization of the sensitivity for vinyl chloride, measured in oxygen mode. The right panel shows a daily calibration check for benzene. The reported concentration, using *k* from Table 2, underestimates the true concentration by about 18% (slope of 0.822). Thus, benzene data for this day are adjusted by the best-fit slope. This process was repeated daily for each species present in our calibration standard.

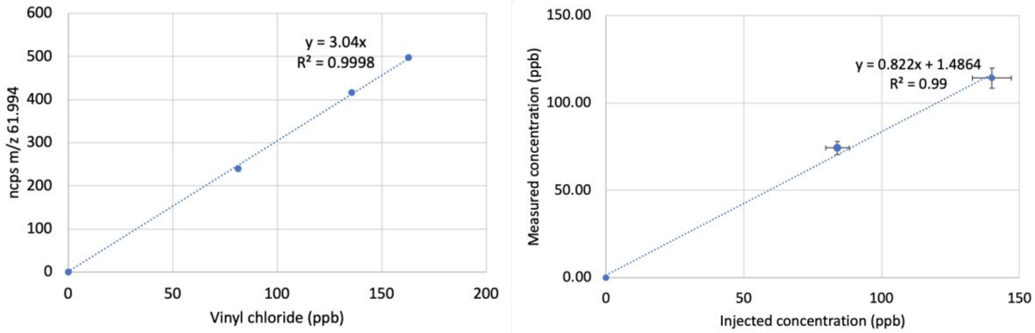

Figure S2. (left) Calibration of vinyl chloride. The resulting sensitivity (3.04 ncps/ppb) was used to convert raw signals to concentration. (right) Calibration check for benzene. There was good linearity, but the reported concentration was biased low by 18%. The resulting data were therefore adjusted based on this bias.

5. Data Interpretation and Visualization

5.1 Acrolein

Because acrolein was not quantified on an absolute scale, we used relative comparison with sampling that was performed in Pittsburgh on 2/16/2023. Specifically, sampling in hydronium mode in “downtown” Pittsburgh occurred from 10:54 am until 11:07 am, and a separate “urban” area of Pittsburgh from 1:22 pm until 1:33 pm. The average value for “downtown” was used as a reference point data from East Palestine data. For mapping, acrolein data (ratio to Pittsburgh mean) within 3 miles of East Palestine (defined as latitude=40.833951 and longitude= -80.540347) were included. Data were averaged over a grid with grid sizes of 150 ft x 150 ft, and log-transformed for color scaling.

5.2. Benzene, Toluene, Vinyl Chloride, and Xylenes

Data for benzene, toluene, vinyl chloride, and xylenes were visualized using violin plots, and compared to available reference levels (Table S3) from the U.S. EPA (Reference Concentrations for lifetime exposure and Concentrations corresponding to 10^{-4} lifetime excess cancer risk) and the ATSDR (Minimal Risk Levels for acute (1 day to 14 days), intermediate (15 days to 1 year), and chronic exposures (>1 year)).

Table S3. Reference Levels for Comparison to Ambient Air Concentrations

Chemical Name (CAS#)	Reference Level Type	Value (μg/m^3)	Source
1,1,2-Trichloroethane (79-00-5)	Chronic RfC	0.2	PPRTV (screening value)
1,3-Butadiene (106-99-0)	Chronic RfC	2	IRIS
Acrolein (107-02-8)	Chronic RfC	0.02	IRIS
Acrolein (107-02-8)	Intermediate MRL	0.09	ATSDR
Benzene (71-43-2)	Chronic RfC	30	IRIS
Benzene (71-43-2)	Chronic MRL	10	ATSDR
Benzene (71-43-2)	Intermediate RfC	19	IRIS
Benzene (71-43-2)	10^{-4} Cancer Risk	13	IRIS
m,p-Xylenes (179601-23-1)	Chronic RfC	100	IRIS
Naphthalene (91-20-3)	Chronic RfC	3	IRIS
o-Xylene (95-47-6)	Chronic RfC	100	IRIS
Toluene (108-88-3)	Chronic RfC	5000	IRIS
Toluene (108-88-3)	Chronic MRL	3800	ATSDR
Trichloroethylene (79-01-6)	Chronic RfC	2	IRIS
Vinyl Chloride (75-01-4)	Chronic RfC	100	IRIS
Vinyl Chloride (75-01-4)	Intermediate MRL	51	ATSDR
Vinyl Chloride (75-01-4)	10^{-4} Cancer Risk	23	IRIS
Xylenes (1330-20-7)	Chronic RfC	100	IRIS
Xylenes (1330-20-7)	Chronic MRL	220	ATSDR
Xylenes (1330-20-7)	Intermediate MRL	2600	ATSDR

ATSDR: Agency for Toxic Substances and Disease Registry; IRIS: Integrated Risk Information System (U.S. EPA); MRL: Minimal Risk Level; PPRTV: Provisional Peer-reviewed Toxicity Values (U.S. EPA); RfC: Reference Concentration.

5.3. Contextual Information

For context, the train manifest is shown in Figure S3, and the meteorological data on the day of sampling is summarized in Figure S4.

LINE #	CAR ID	LOAD/MTY	CAR TYPE	COMMODITY	TANK CAR SPEC	UN ID	HAZ CLASS	Status of Car
23	ARSX 4145	LOADED	HOPPER	POLYPROPYLENE				Not in derailment pile
24	BRKX 66738	LOADED	HOPPER	POLYPROPYLENE				Not in derailment pile
25	GPLX 75465	LOADED	HOPPER	POLYETHYLENE				lading destroyed by fire
26	ECUX 860375	LOADED	HOPPER	POLYETHYLENE				lading destroyed by fire
27	UTLX 684543	EMPTY	TANK CAR	residue lube oil	DOT 117J100W			scrap pending C&P
28	TILX 402025	LOADED	TANK CAR	VINYL CHLORIDE, STABILIZED	DOT 105J300W	UN1086	2.1 (FLAMMABLE GAS)	car did not leak/cars vent product through the PRD and ignited/vent and burn performed
29	OCPX 80235	LOADED	TANK CAR	VINYL CHLORIDE, STABILIZED	DOT 105J300W	UN1086	2.1 (FLAMMABLE GAS)	car did not leak/cars vent product through the PRD and ignited/vent and burn performed
30	OCPX 80179	LOADED	TANK CAR	VINYL CHLORIDE, STABILIZED	DOT 105J300W	UN1086	2.1 (FLAMMABLE GAS)	car did not leak/cars vent product through the PRD and ignited/vent and burn performed
31	GATX 95098	LOADED	TANK CAR	VINYL CHLORIDE, STABILIZED	DOT 105J300W	UN1086	2.1 (FLAMMABLE GAS)	vent product through the PRD and ignited/vent and burn
32	RACX 51629	LOADED	TANK CAR	DIPROPYLENE GLLYCOL	DOT 111A100W1			fire impingement/no signs of tank breach
33	LYBX 5191	LOADED	TANK CAR	PROPYLENE GLYCOL	DOT 117J100W			flame impingement, no tank breach found
34	RACX 51435	LOADED	TANK CAR	PROPYLENE GLYCOL	DOT 111A100W1			tank breached/lost most of load
35	UTLX 671772	LOADED	TANK CAR	DIETHYLENE GLYCOL	DOT 111A100W1			had small leak from BOV, unknown amount of product in car
36	SHPX 211226	LOADED	TANK CAR	COMBUSTIBLE LIQ., NOS (ETHYLENE GLYCOL MONOBUTYL ETHER)	DOT 111S100W1	NA1993	COMBUSTIBE LIQUID	unknown status
37	TILX 331319	LOADED	HOPPER	SEMOLINA				in pile, destroyed by fire
38	DOWX 73168	LOADED	TANK CAR	COMBUSTIBLE LIQ., NOS (ETHYLHEXYL ACRYLATE)	DOT 111S100W1	NA1993	COMBUSTIBE LIQUID	Car breached on head end/amount of product still in car pending
39	ROIX 57036	LOADED	HOPPER	POLYVINYL				burned
40	NCUX 40057	LOADED	HOPPER	POLYVINYL				actively burning
41	UTLX 100055	LOADED	TANK CAR	PETROLEUM LUBE OIL	DOT 111A100W1			double comp car/both breached/entire load lost
42	XOMX 110664	LOADED	TANK CAR	PETROLEUM LUBE OIL	211A100W1			tank breached/lost most of load
43	UTLX 684798	LOADED	TANK CAR	PETROLEUM LUBE OIL	DOT 117J100W			flame impinged, may have had a small leak/will be determined when car is off loaded
44	UTLX 671310	LOADED	TANK CAR	PETROLEUM LUBE OIL	DOT 111A100W1			flame impinged, small leak from top fittings, unknown amount left in tank
45	CERX 30072	LOADED	TANK CAR	POLYPROPYL GLYCOL	DOT 111A100W1			flame impinged, tank breached/ most of load lost
46	SHPX 211106	LOADED	TANK CAR	PROPYLENE GLYCOL	DOT 111S100W1			flame impinged, no signs of breach
47	NATX 231335	LOADED	TANK CAR	DIETHYLENE GLYCOL	DOT 111A100W1			flame impinged, tank breached/ load lost
48	UTLX 671913	LOADED	TANK CAR	DIETHYLENE GLYCOL	DOT 111A100W1			flame impinged, lost unknown amount at this time from damaged BOV
49	NATX 35844	LOADED	TANK CAR	ISOBUTYLENE	DOT 105J300W	UN1055	2.1 (FLAMMABLE GAS)	some flame impingement/no signs of breach
50	UTLX 205907	LOADED	TANK CAR	BUTYL ACRYLATES, STABILIZED	DOT 111A100W1	UN 2348	3 (FLAMMABLE LIQUID)	Head breach/lost entire load (spill& fire)
51	UTLX 661296	LOADED	TANK CAR	PETRO OIL, NEC	DOT 111A100W1			flame impinged, small leak from VRV stopped, car still loaded

Figure S3. Train manifest, as provided by Norfolk Southern, with chemicals of concern highlighted.

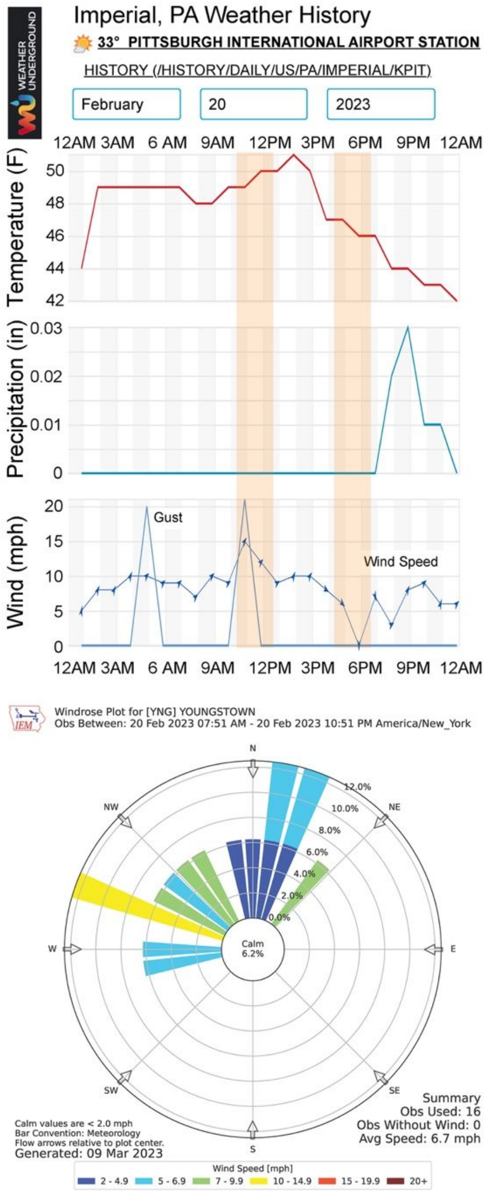

Figure S4. Summary of temperature, precipitation, wind speed, and wind direction at nearest weather station to East Palestine (Pittsburgh International Airport Station) on the day of sampling (February 20, 2023). Source: Weather Underground.

References

(1) Lindinger, W.; Hansel, A.; Jordan, A. On-Line Monitoring of Volatile Organic Compounds at Pptv Levels by Means of Proton-Transfer-Reaction Mass Spectrometry (PTR-MS) Medical Applications, Food Control and Environmental Research. *Int J Mass Spectrom Ion Process* **1998**, *173* (3), 191–241. https://doi.org/10.1016/S0168-1176(97)00281-4.

(2) Pagonis, D.; Sekimoto, K.; de Gouw, J. A Library of Proton-Transfer Reactions of H3O+ Ions Used for Trace Gas Detection. *J Am Soc Mass Spectrom* **2019**, *30* (7), 1330–1335. https://doi.org/10.1007/S13361-019-02209-3.

(3) Sekimoto, K.; Li, S. M.; Yuan, B.; Koss, A.; Coggon, M.; Warneke, C.; de Gouw, J. Calculation of the Sensitivity of Proton-Transfer-Reaction Mass Spectrometry (PTR-MS) for Organic Trace Gases Using Molecular Properties. *Int J Mass Spectrom* **2017**, *421*, 71–94. https://doi.org/10.1016/J.IJMS.2017.04.006.

(4) de Gouw, J.; Warneke, C. Measurements of Volatile Organic Compounds in the Earth's Atmosphere Using Proton-Transfer-Reaction Mass Spectrometry. *Mass Spec. Rev.* **2007**, *26* (2), 223–257.

(5) Brilli, F.; Gioli, B.; Ciccioli, P.; Zona, D.; Loreto, F.; Janssens, I. A.; Ceulemans, R. Proton Transfer Reaction Time-of-Flight Mass Spectrometric (PTR-TOF-MS) Determination of Volatile Organic Compounds (VOCs) Emitted from a Biomass Fire Developed under Stable Nocturnal Conditions. *Atmos Environ* **2014**, *97*, 54–67. https://doi.org/10.1016/J.ATMOSENV.2014.08.007.

(6) Schieweck, A.; Uhde, E.; Salthammer, T. Determination of Acrolein in Ambient Air and in the Atmosphere of Environmental Test Chambers. *Environ Sci Process Impacts* **2021**, *23* (11), 1729–1746. https://doi.org/10.1039/D1EM00221J.

(7) Koss, A. R.; Sekimoto, K.; Gilman, J. B.; Selimovic, V.; Coggon, M. M.; Zarzana, K. J.; Yuan, B.; Lerner, B. M.; Brown, S. S.; Jimenez, J. L.; Krechmer, J.; Roberts, J. M.; Warneke, C.; Yokelson, R. J.; De Gouw, J. Non-Methane Organic Gas Emissions from Biomass Burning: Identification, Quantification, and Emission Factors from PTR-ToF during the FIREX 2016 Laboratory Experiment. *Atmos Chem Phys* **2018**, *18* (5), 3299–3319. https://doi.org/10.5194/ACP-18-3299-2018.

(8) Stockwell, C. E.; Veres, P. R.; Williams, J.; Yokelson, R. J. Characterization of Biomass Burning Emissions from Cooking Fires, Peat, Crop Residue, and Other Fuels with High-Resolution Proton-Transfer-Reaction Time-of-Flight Mass Spectrometry. *Atmos Chem Phys* **2015**, *15* (2), 845–865. https://doi.org/10.5194/ACP-15-845-2015.
